# Electronic Structure of C_60_/Zinc Phthalocyanine/V_2_O_5_ Interfaces Studied Using Photoemission Spectroscopy for Organic Photovoltaic Applications

**DOI:** 10.3390/molecules23020449

**Published:** 2018-02-18

**Authors:** Chang Jin Lim, Min Gyu Park, Min Su Kim, Jeong Hwa Han, Soohaeng Cho, Mann-Ho Cho, Yeonjin Yi, Hyunbok Lee, Sang Wan Cho

**Affiliations:** 1 Department of Physics, Yonsei University, 1 Yonseidae-gil, Wonju-si, Gangwon-do 26493, Korea; eyepiece89@yonsei.ac.kr (C.J.L.); ham744@naver.com (M.G.P.); shcho@yonsei.ac.kr (S.C.); 2 Institute of Physics and Applied Physics, Yonsei University, 50 Yonsei-ro, Seodaemun-Gu, Seoul 03722, Korea; kimssu98k@yonsei.ac.kr (M.S.K.); jh-han@yonsei.ac.kr (J.H.H.); mh.cho@yonsei.ac.kr (M.-H.C.); yeonjin@yonsei.ac.kr (Y.Y.); 3 Department of Physics, Kangwon National University, 1 Gangwondaehak-gil, Chuncheon-si, Gaongwon-do 24341, Korea; hyunbok@kangwon.ac.kr

**Keywords:** organic photovoltaics, photoemission spectroscopy, energy band diagram, ZnPc, V_2_O_5_

## Abstract

The interfacial electronic structures of a bilayer of fullerene (C_60_) and zinc phthalocyanine (ZnPc) grown on vanadium pentoxide (V_2_O_5_) thin films deposited using radio frequency sputtering under various conditions were studied using X-ray and ultraviolet photoelectron spectroscopy. The energy difference between the highest occupied molecular orbital (HOMO) level of the ZnPc layer and the lowest unoccupied molecular orbital (LUMO) level of the C_60_ layer was determined and compared with that grown on an indium tin oxide (ITO) substrate. The energy difference of a heterojunction on all V_2_O_5_ was found to be 1.3~1.4 eV, while that on ITO was 1.1 eV. This difference could be due to the higher binding energy of the HOMO of ZnPc on V_2_O_5_ than that on ITO regardless of work functions of the substrates. We also determined the complete energy level diagrams of C_60_/ZnPc on V_2_O_5_ and ITO.

## 1. Introduction

Organic photovoltaics (OPVs) have received increasing attention over the past few years due to their potential as a renewable, cheap, and economical source of power [1,2,3,4,5,6]. However, despite significant recent advances in their cell performance, the current power conversion efficiencies remain too low for commercial viability of such cells. A general problem of organic electronic devices is the poor transport of charge carriers at the interface between electrodes and the organic semiconductor. Consequently, recent efforts have been made to improve charge transport and collection at the electrodes. In particular, the use of transition metal oxides as a transparent electrode has attracted considerable interest [7]. Indium tin oxide (ITO) is widely used as an electrode in OPV fabrication and as a transparent conducting electrode for light-emitting diodes due to its reasonable transparency in the visible region, good conductivity, and ease of patterning. However, the surface chemistry of ITO is difficult to control [8] and ITO has become approximately 10 times more expensive over the past few years due to diminishing indium resources [9]. In fact, the cost of indium is expected to increase because of the increasing demand from producers of solar cells in addition to the demand from the existing flat-panel display industry [10]. Transition metal oxides are believed to prevent unwanted chemical reactions between transparent electrodes and an optically active organic layer [11,12,13]. Furthermore, a high work function material such as a metal oxide is desirable to decrease the series resistance in devices [14]. Due to different oxidation states, vanadium can be used to create many compounds with oxygen such as vanadium monoxide (VO), vanadium sesquioxide (V_2_O_3_), vanadium dioxide (VO_2_), vanadium pentoxide (V_2_O_5_), V_3_O_7_, V_6_O_13_, and V_4_O_9_ [15], which exhibit specific properties depending on structure. At a critical temperature, crystallographic transformation shows that vanadium oxide accompanies a reversible semiconductor-to-metal transition that alters its optical and electrical properties [16]. Among the possible compounds of vanadium oxides, the stable V_2_O_5_ phase possesses a wide optical band gap, good chemical and thermal stability, and excellent thermoelectric properties, which suggest that V_2_O_5_ is a potential candidate in several applications including electro-chromic displays, gas sensing, and optoelectronic devices [17,18,19,20]. Different deposition techniques such as spray pyrolysis, ultrasonic spray pyrolysis, spin coating, thermal evaporation, electron beam evaporation, radio frequency sputtering, dc magnetron sputtering, sol-gel, and chemical vapor deposition have been used to deposit vanadium oxide thin films in order to improve the optical and electrical properties of transparent conductive oxides (TCOs).

In this study, the electronic structures and energy level alignment of a bilayer of C_60_ and ZnPc grown on V_2_O_5_ thin films deposited using radio frequency sputtering under various conditions were studied. The electronic structure was measured using in-situ ultraviolet photoemission spectroscopy (UPS) and X-ray photoemission spectroscopy (XPS). The separation of the highest occupied molecular orbital (HOMO) of the donor and the lowest unoccupied molecular orbital (LUMO) of the acceptor (E^D^_HOMO_–E^A^_LUMO_) was measured for each bilayer because E^D^_HOMO_–E^A^_LUMO_ is a strong factor in determining the magnitude of the measured open circuit voltage (V_OC_) of an OPV cell based on a donor–acceptor heterojunction [21,22].

## 2. Results and Discussion

As shown in Table 1, V_2_O_5_ samples were fabricated under three different growth conditions. The V_2_O_5_ #1 sample was grown in an Ar atmosphere for 5 min, and the V_2_O_5_ #2 sample was grown in an Ar atmosphere for 20 min. Alternatively, the V_2_O_5_ #3 sample was deposited in an Ar:O2 atmosphere (at a ratio of 29:1) for 20 min. The sheet resistances of the samples were not significantly different.

**Figure 1:**
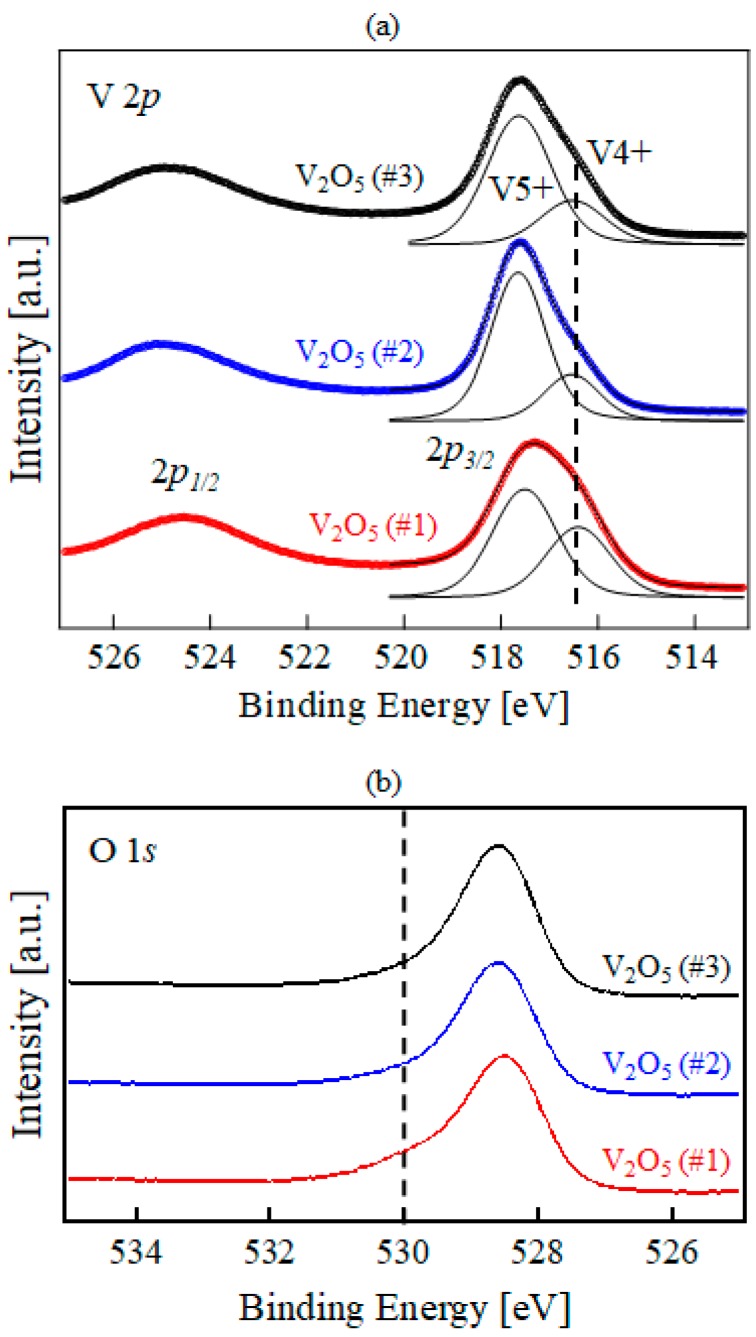
(**a**) V 2*p* and (**b**) O 1*s* core level photoemission spectra of V_2_O_5_ #1, V_2_O_5_ #2, and V_2_O_5_ #3, measured with a monochromatic Al Ka X-ray source.

To determine the chemical state of the elements in the obtained samples, XPS analysis was carried out. These results are shown in Figure 1. For vanadium, a complex energy distribution of V 2*p* photoelectrons was obtained. The V 2*p* core level spectrum is dominated by a spin-orbit doublet with peaks at binding energies of about 517.5 and 525.0 eV. Both the binding energy and spin-orbit splitting are in good agreement with values previously reported for V_2_O_5_ [23]. The line shapes of these spectra were analyzed by a standard least squares fitting scheme using a convolution of Gaussian and Lorentzian peaks. The width of the Lorentzian peaks was assumed to be the same for each component. We obtain an fwhm for the Lorentzian of about 0.35 eV for V 2*p*_3/2_. The FWHM for the Gaussian resulting from the fitting procedure is 1.20~1.40 eV. As shown in Figure 1a, an additional peak in the V 2*p*_3/2_ core level was also observed at lower binding energy (about 516.4 eV) in all samples; however, the relative intensity of that in V_2_O_5_ #1 was greater than in the others. The peak position is close to that reported for single crystalline VO_2_ [23]. The oxidation states of vanadium V_2_O_5_ and VO_2_ are V^5+^ and V^4+^, respectively. This means that the V_2_O_5_ #1 sample did not have enough deposition time to form a perfect composition and had relatively more oxygen vacancies, which account for the higher defect level and greater abundance of trap-assisted conducting oxides than in the others. In Figure 1b, an additional O 1*s* core level was also observed at higher binding energies in all samples. The relative intensity of that of V_2_O_5_ #1 is also greater than the others in the O 1*s* core level spectra.

**Figure 2:**
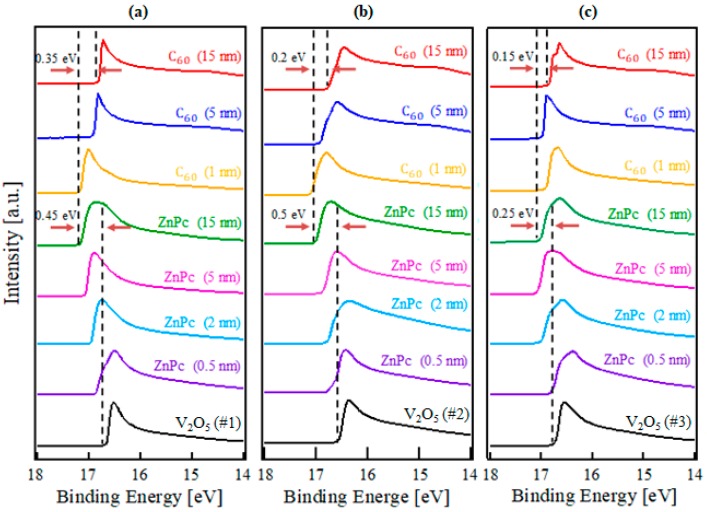
The UPS spectra in the secondary cut-off region collected during the step-by-step layer deposition of C_60_/ZnPc on (**a**) V_2_O_5_ #1, (**b**) V_2_O_5_ #2, and (**c**) V_2_O_5_ #3 surfaces.

**Figure 3:**
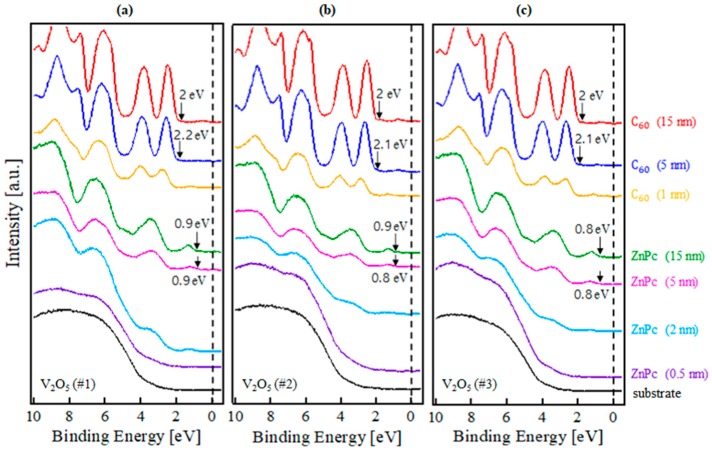
The UPS spectra collected near the Fermi level as a function of the C_60_/ZnPc deposition thickness on (**a**) V_2_O_5_ #1, (**b**) V_2_O_5_ #2, and (**c**) V_2_O_5_ #3 surfaces.

The electronic structures during the interface formation of C_60_/ZnPc/V_2_O_5_ #1, #2, and #3 were investigated using in-situ UPS. Figure 2 and Figure 3 show UPS spectra obtained for the deposition of C_60_/ZnPc on various V_2_O_5_ thin films. The spectra shown in Figure 2a–c were collected in the secondary electron cut-off region for the V_2_O_5_ #1, V_2_O_5_ #2, and V_2_O_5_ #3 layers, respectively. The supplementary material (Figure S1) illustrates the procedure used for the determination of energy-level alignment at the interface. The secondary cut-off position moved noticeably toward a higher binding energy as soon as the ZnPc deposition began on each substrate. The shift of the secondary cut-off position is attributed to the formation of an interface dipole and band bending [24,25]. However, the secondary cut-off position moved toward lower binding energies as soon as C_60_ deposition began on each ZnPc layer. In these spectra, the total shift of the cut-off position of ZnPc toward higher binding energies was 0.45, 0.50, and 0.25 eV, while that of C_60_ on the ZnPc layer was 0.35, 0.20, and 0.15 eV toward lower binding energies for V_2_O_5_ #1, V_2_O_5_ #2, and V_2_O_5_ #3, respectively.

Figure 3a–c show the evolution of HOMO onset during the growth of the C_60_/ZnPc layer on V_2_O_5_ #1, V_2_O_5_ #2, and V_2_O_5_ #3, respectively. As shown in Figure 3a, there was no shift in HOMO onset of ZnPc for the ZnPc/V_2_O_5_ #1 interface and the HOMO onset was measured at 0.9 eV below the Fermi level. It is clear that the HOMO of C_60_ gradually shifted toward lower binding energies, and total shift reached 0.2 eV after the shifts were saturated. This result confirms that band bending occurred at the C_60_/ZnPc interface. The saturated HOMO onset of the C_60_ layer was 2.0 eV below the Fermi level. In Figure 3b, the HOMO onset of ZnPc gradually shifted toward higher binding energies from 0.8 to 0.9 eV, confirming band bending at the ZnPc/V_2_O_5_ #2 interface. It was also observed that the HOMO of C_60_ gradually shifted toward lower binding energies, and the total shift reached 0.1 eV. The saturated HOMO onset of the C_60_ layer was 2.0 eV below the Fermi level. In Figure 3c, the HOMO onset of ZnPc on V_2_O_5_ #3 did not shift, and the HOMO onset was measured at 0.8 eV below the Fermi level. The HOMO of C_60_ shifted toward lower binding energies from 2.1 to 2.0 eV.

**Figure 4:**
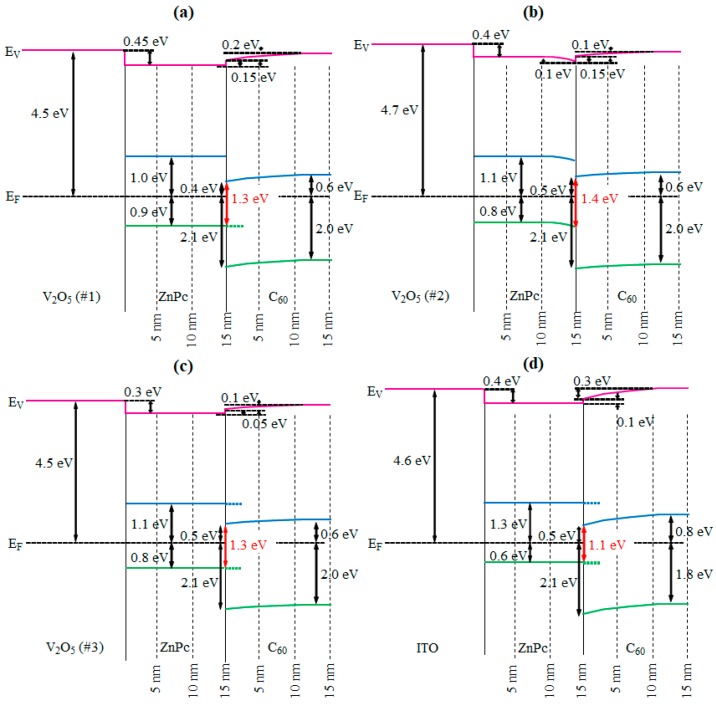
Energy level diagrams of C_60_/ZnPc on (**a**) V_2_O_5_ #1, (**b**) V_2_O_5_ #2, (**c**) V_2_O_5_ #3, and (**d**) ITO surfaces.

An energy level diagram was constructed by combining the changes of the spectra shown in Figure 2 and Figure 3. We estimate that the overall measurement errors in orbital offsets and interface dipoles are of the order of ±0.05 eV. We determined the energy positions according to the systematic procedure as described in detail in [7]. Additionally, the energy level diagram of C_60_/ZnPc/ITO based on our data (not shown here) is displayed. As shown in Figure 4, the energy gaps between the HOMO and LUMO of ZnPc and C_60_ were about 1.9 and 2.6 eV, respectively, as previously reported [26,27]. The measured work functions of V_2_O_5_ #1, V_2_O_5_ #2, and V_2_O_5_ #3 were estimated to be about 4.5, 4.7, and 4.5 eV, respectively. The energy level difference between the HOMO of the donor (ZnPc) and the LUMO of the acceptor (C_60_), E^D^_HOMO_–E^A^_LUMO_ is an important factor in determining the magnitude of V_OC_ of OPVs based on a donor–acceptor heterojunction. However, a correction term between the real V_OC_ and the E^D^_HOMO_–E^A^_LUMO_ value may be required to account for voltage losses in the device due to large diode quality factors, high reverse saturation currents, low-field-dependent mobilities of charge carriers, and voltage losses at the collection electrodes.

The C_60_/ZnPc heterojunction on V_2_O_5_ #2 showed the largest E^D^_HOMO_–E^A^_LUMO_ value (1.4 eV). The measured E^D^_HOMO_–E^A^_LUMO_ values on V_2_O_5_ #1 and V_2_O_5_ #3 were the same (1.3 eV). There are several factors that can affect the magnitude of E^D^_HOMO_–E^A^_LUMO_ at the donor–acceptor interface, including the ionization potential of a donor, the electron affinity of an acceptor, the formation of interface dipoles, and charge redistribution across the interfaces. In this case, the band bending of ZnPc was observed only at the ZnPc/V_2_O_5_ #2 interface, and the band bending moved the HOMO of the donor (ZnPc) toward higher binding energies and increased the E^D^_HOMO_–E^A^_LUMO_ value. In general, this is due to different degrees of charge transfer from the substrate to the ZnPc layer, and the work function difference of substrates is the origin of the different degrees of charge transfer. However, the value of band bending (0.1 eV) was almost negligible considering the measurement error. Therefore, this 0.1 eV difference of the E^D^_HOMO_–E^A^_LUMO_ value was not predicted to lead to enhanced values of V_OC_ for OPVs. On the other hand, the measured E^D^_HOMO_–E^A^_LUMO_ values on ITO was 1.1 eV, which is smaller than those on V_2_O_3_ samples. This means that the OPV using V_2_O_3_ as an anode should have a larger V_OC_ than that using ITO. This is because the HOMO onset of ZnPc on ITO was 0.2~0.3 eV lower than that on the V_2_O_5_ regardless of work functions of the substrates. To understand the underlying cause, further studies should be conducted comparing the charge neutrality level of ZnPc with the density of states of V_2_O_5_ and ITO. Therefore, not only the work functions of new TCOs but also the charge neutrality level of donors should be considered for controlling the V_OC_ for high-efficiency OPVs.

## 3. Methods

V_2_O_5_ films on ITO substrate were prepared by radio frequency magnetron sputtering of V_2_O_5_ targets at room temperature. Before deposition the ITO substrates were sequentially cleaned ultrasonically in acetone, alcohol, and de-ionized water before finally being dried in nitrogen gas. The distance between the target and substrate was approximately 100 mm. The radio frequency magnetron working power was 200 W, and deposition times were 5 and 20 min. The base pressure was 5.7 × 10^−7^ Torr and the working pressure was 2.5 m Torr. As shown in Table 1, three V_2_O_5_ films at different deposition times and flowing gas compositions were prepared.

The chemical states for the V_2_O_5_ films grown on ITO were examined by XPS. XPS core level spectra of V 2*p* and O 1*s* were obtained using a monochromatic Al Ka X-ray source. In situ UPS measurements were carried out using a PHOIBOS 150 energy analyzer (SPECS GmbH, Berlin, Germany) and an ultraviolet (He I, 21.22 eV) light source. The base pressure of the analysis chamber was 5 × 10^−10^ Torr. ZnPc and C_60_ were thermally evaporated on V_2_O_5_ and ITO substrates at a rate of 0.01 nm/s. The total thickness and deposition rate were monitored by a quartz crystal microbalance. The secondary electron cut-off (SEC) positions were measured in normal emission geometry with a sample bias of −5 V.

## 4. Conclusions

The interfacial electronic structures of a bilayer of C_60_ and ZnPc grown on V_2_O_5_ thin films deposited using radio frequency sputtering under various conditions were studied using X-ray and ultraviolet photoelectron spectroscopy. The energy difference between the HOMO level of the ZnPc layer and the LUMO level of the C_60_ layer was determined and compared with that grown on ITO substrate. The energy difference of a heterojunction on all V_2_O_5_ was found to be 1.3~1.4 eV, while that on ITO was 1.1 eV. This difference could be due to the HOMO of ZnPc on V_2_O_5_ having a higher binding energy than that on ITO regardless of work functions of the substrates. We also determined the complete energy level diagrams of C_60_/ZnPc on V_2_O_5_ and ITO.

## Figures and Tables

**Table 1:** The growth conditions of V_2_O_5_ #1, #2, and #3.

**V_2_O_5_ #1**	
Growth time (min)	5
Gas flow (sccm)	Ar = 30
Sheet resistivity (Ω/sq.)	48
**V_2_O_5_ #2**	
Growth time (min)	20
Gas flow (sccm)	Ar = 30
Sheet resistivity (Ω/sq.)	46
**V_2_O_5_ #3**	
Growth time (min)	20
Gas flow (sccm)	Ar:O2 = 29:1
Sheet resistivity (Ω/sq.)	36

## Supplementary Materials

The Supplementary Materials are available online.
