# Supplementary material for: Electronic Structure of C60/Zinc Phthalocyanine/V2O5 Interfaces Studied Using Photoemission Spectroscopy for Organic Photovoltaic Applications
Source: Molecules. 2018 Feb 18;23(2):449. doi: 10.3390/molecules23020449 (PMC6017094; doi:10.3390/molecules23020449)
Supplement: Supplementary file 1 [file molecules-23-00449-s001.pdf]

Supporting Information for:

Electronic structure of C_{60}/zinc phthalocyanine/V_2O_5 interfaces studied using photoemission spectroscopy for organic photovoltaic applications

Chang Jin Lim[a], Min Gyu Park[a], Min Su Kim[b], Jeong Hwa Han[b], Soohaeng Cho[a], Mann-Ho Cho[b], Yeonjin Yi[b], Hyunbok Lee[c], and Sang Wan Cho[a,*]

[a] Department of Physics, Yonsei University, 1 Yonseidae-gil, Wonju-si, Gangwon-do 26493 Korea

[b] Institute of Physics and Applied Physics, Yonsei University, 50 Yonsei-ro, Seodaemun-Gu, Seoul 03722, Republic of Korea

[c] Department of Physics, Kangwon National University, 1 Gangwondaehak-gil, Chuncheon-si, Gaongwon-do 24341, Republic of Korea.

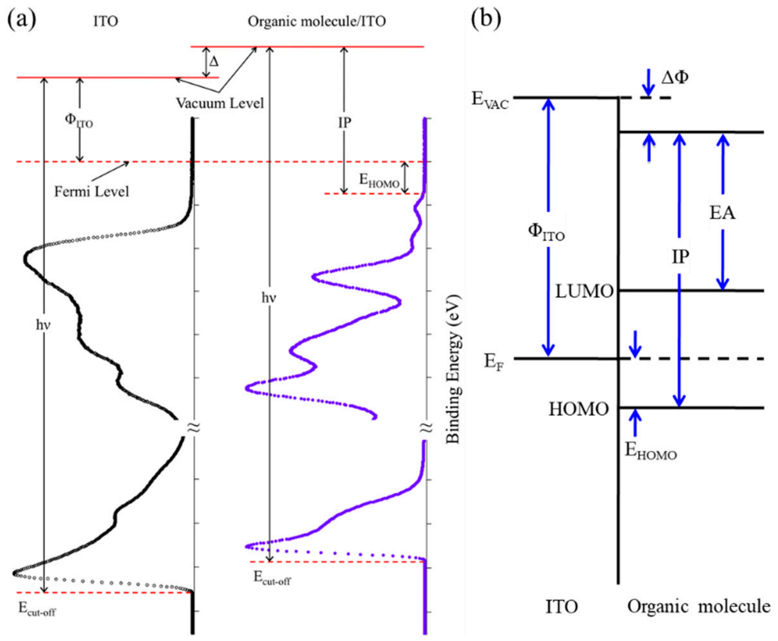

Figure S1. (a) Schematic illustration of some of the important parameters derived from PES characterization of surfaces and interfaces. (b) An energy-level diagram for a generic junction formed between an organic film and an ITO substrate.

Figure S1 illustrates the procedure used for the determination of energy-level alignment at the interface. The basic equation used in interpreting photoelectron spectra is:

$$E_B = h\nu - E_k - \Phi \quad (1)$$

The photon energy (hν) is known and the photoelectron kinetic energy (E_k) is measured in order to deduce the binding energy (E_B) referenced to E_F. When hν is known, the work function (Φ) can be obtained from the measured energy of the secondary-electron cut-off ($E_{cut-off}$)., i.e.:

$$\Phi = h\nu - E_{cut-off} \quad (2)$$

The change in the work function, ΔΦ, can then be tracked by measuring $E_{cut-off}$ after a deposition step. Therefore, the shift of this $E_{cut-off}$ indicates the magnitude of the interfacial dipole, which is equal to increasing or decreasing the work function. Similarly, the ionization potential (IP) can be obtained from $E_{cut-off}$ and the HOMO onset (E_{HOMO}):

$$IP = h\nu - (E_{cut-off} - E_{HOMO}) \quad (3)$$
